# Users’ Experiences With Online Access to Electronic Health Records in Mental and Somatic Health Care: Cross-Sectional Study

**DOI:** 10.2196/47840

**Published:** 2023-12-25

**Authors:** Bo Wang, Eli Kristiansen, Asbjørn Johansen Fagerlund, Paolo Zanaboni, Maria Hägglund, Annika Bärkås, Sari Kujala, Åsa Cajander, Charlotte Blease, Anna Kharko, Isto Huvila, Bridget Kane, Monika Alise Johansen

**Affiliations:** 1 Norwegian Centre for E-health Research University Hospital of North Norway Tromsø Norway; 2 Department of Clinical Medicine UiT The Arctic University of Norway Tromsø Norway; 3 Participatory eHealth and Health Data Research Group Department of Women's and Children's Health Uppsala University Uppsala Sweden; 4 Medtech Science & Innovation Centre Uppsala University Hospital Uppsala Sweden; 5 Department of Computer Science Aalto University Espoo Finland; 6 Department of Information Technology Uppsala University Uppsala Sweden; 7 Digital Psychiatry, Department of Psychiatry Beth Israel Deaconess Medical Center Harvard Medical School Boston, MA United States; 8 Faculty of Health University of Plymouth Plymouth United Kingdom; 9 Department of Archives, Libraries & Museums Uppsala University Uppsala Sweden; 10 Business School Karlstad University Karlstad Sweden

**Keywords:** patient empowerment, online access to electronic health records, patient-accessible electronic health record, patient access, user perspective, psychiatry, electronic health record, health data, patient portal, online records access

## Abstract

**Background:**

Patient-accessible electronic health records (PAEHRs) hold promise for empowering patients, but their impact may vary between mental and somatic health care. Medical professionals and ethicists have expressed concerns about the potential challenges of PAEHRs for patients, especially those receiving mental health care.

**Objective:**

This study aims to investigate variations in the experiences of online access to electronic health records (EHRs) among persons receiving mental and somatic health care, as well as to understand how these experiences and perceptions vary among those receiving mental health care at different levels of point of care.

**Methods:**

Using Norwegian data from the NORDeHEALTH 2022 Patient Survey, we conducted a cross-sectional descriptive analysis of service use and perceptions of perceived mistakes, omissions, and offensive comments by mental and somatic health care respondents. Content analysis was used to analyze free-text responses to understand how respondents experienced the most serious errors in their EHR.

**Results:**

Among 9505 survey participants, we identified 2008 mental health care respondents and 7086 somatic health care respondents. A higher percentage of mental health care respondents (1385/2008, 68.97%) reported that using PAEHR increased their trust in health care professionals compared with somatic health care respondents (4251/7086, 59.99%). However, a significantly larger proportion (*P*<.001) of mental health care respondents (976/2008, 48.61%) reported perceiving errors in their EHR compared with somatic health care respondents (1893/7086, 26.71%). Mental health care respondents also reported significantly higher odds (*P*<.001) of identifying omissions (758/2008, 37.75%) and offensive comments (729/2008, 36.3%) in their EHR compared with the somatic health care group (1867/7086, 26.35% and 826/7086, 11.66%, respectively). Mental health care respondents in hospital inpatient settings were more likely to identify errors (398/588, 67.7%; *P*<.001) and omissions (251/588, 42.7%; *P*<.001) than those in outpatient care (errors: 422/837, 50.4% and omissions: 336/837, 40.1%; *P*<.001) and primary care (errors: 32/100, 32% and omissions: 29/100, 29%; *P*<.001). Hospital inpatients also reported feeling more offended (344/588, 58.5%; *P*<.001) by certain content in their EHR compared with respondents in primary (21/100, 21%) and outpatient care (287/837, 34.3%) settings. Our qualitative findings showed that both mental and somatic health care respondents identified the most serious errors in their EHR in terms of medical history, communication, diagnosis, and medication.

**Conclusions:**

Most mental and somatic health care respondents showed a positive attitude toward PAEHRs. However, mental health care respondents, especially those with severe and chronic concerns, expressed a more critical attitude toward certain content in their EHR compared with somatic health care respondents. A PAEHR can provide valuable information and foster trust, but it requires careful attention to the use of clinical terminology to ensure accurate, nonjudgmental documentation, especially for persons belonging to health care groups with unique sensitivities.

## Introduction

Patient-accessible electronic health records (PAEHRs) are online services that allow patients to view and sometimes edit or comment on their electronic health records (EHRs), including clinical assessments, laboratory results, radiology findings, nursing documentation, allergy information, medication information, and discharge letters [1-3]. A PAEHR has the potential to improve medical care in various areas, particularly by enhancing patient empowerment [4] and patient-provider communication [5-7]. Studies from the Nordic countries and the United States have reported that service users are generally positive toward PAEHR. According to a survey study by Moll et al [8] in 2018, more than 96% of the respondents (n=2541) expressed a favorable attitude toward the Swedish national health portal Journalen, with most of them believing that online access to EHRs improved self-care and communication with medical staff. Similarly, in Norway, a survey by Zanaboni et al [4] in 2019 found that patients were satisfied with PAEHR as it improved their confidence in understanding their health status and helped them prepare for appointments. Patients also felt more secure in reporting mistakes and omissions at the next visit, which was particularly important for those with complex and long-term conditions [4]. In the Unites States, Walker et al [9] in 2019 found that after 7 years of online access to open notes and patient-accessible consultation notes written by a clinician, in outpatient care, approximately two-thirds of the patients described the service as extremely important in increasing their sense of control and taking care of their health.

However, concerns have been raised by health care professionals, especially mental health care (MHC) professionals and medical ethicists [10-12], regarding the transparency of information intended for medical professionals being made available to patients, as it could trigger unnecessary anxiety, distress, or confusion [6,13]. Patients who received MHC report feeling stigmatized [14] and information and terminology can be intrinsically more sensitive than general medical information [6], and the readers could find certain formulations, such as *chronic schizophrenic*, more offensive than others, such as *chronic diabetic* [5,15]. Previous studies have found that psychiatrists and psychologists hold more negative attitudes toward open mental health notes than psychiatric care nurses [16,17], expressing fear of additional workplace burden [15-21], spending more time responding to patient anxieties [13,15-18,22], and spending less time on direct patient care [23]. MHC professionals in Norway have expressed concern about whether PAEHR is suitable and safe for the most susceptible patients [23,24]. Some have even reported writing shadow records due to fears of unexpected outcomes from the service [23,24]. Aside from shadow records, a further concern is that clinicians may simplify their note-taking in an effort to make them more acceptable or understandable to patients, which may compromise the quality of the records [25]. One study from the United States suggested that some clinicians change their language and how they write differential diagnoses with the knowledge that patients will read what they write [26].

In contrast, early studies support the idea that patients with mental health conditions may accrue benefits from PAEHR. One secondary survey analysis in the United States [27] compared the experience with PAEHR among patients with serious mental health diagnoses (defined as major depressive, psychotic, schizoaffective, or bipolar-related disorders) with those with other mental health diagnoses and no mental health diagnoses. The study found that 20% of patients with serious mental health diagnoses and 18% with other mental health diagnoses reported that they were more likely to adhere to their medications after reading their notes, compared with 14% of persons with no mental health diagnosis [27]. Patients with severe mental health diagnoses were also more likely to report that access strengthened their understanding of why medications were prescribed (67%) and helped answer questions about their medications (60%) [27].

In Norway, PAEHRs have been implemented in 3 of the 4 health regions. Northern Norway, Western Norway, and South-Eastern Norway have been offering patients access to their EHRs from hospitals through the national health portal [28] since 2015, 2017, and 2019, respectively [29]. EHRs from general practitioners, dentists, and other specialists are not yet available digitally. A pilot implementation of PAEHR was introduced in Central Norway in 2022, but widespread PAEHR is not yet available in this region [30]. Currently, PAEHR is offered to patients aged ≥16 years and to parents of children below the age of 12 years. Hospital documents such as medical notes, referral letters, outpatient visit summaries, psychiatric reports, and discharge notes are made available online after being approved by health care professionals [5].

Although Norway’s implementation of PAEHR in MHC is similar to that of general health care in terms of timeline, the impact of PAEHR can differ significantly between persons who received mental and somatic health care [13,31]. Despite the ongoing debate on this subject, there is still limited evidence regarding PAEHR user experiences among patients with mental health conditions [13]. Furthermore, there is a lack of research comparing the experiences and perceptions of both mental health and somatic patients with online access to EHR. To address this gap, we conducted a study to investigate the following questions: (1) Are there differences in the use of PAEHR between persons who have received MHC and those who have received somatic health care? (2) Are there differences in the perception of errors, omissions, and offending comments within PAEHR between persons who have received MHC and those who have received somatic health care, and what are the most serious errors reported by both health care groups? (3) Are there differences in experiences with the use of PAEHR among persons who have received MHC across different levels of point of care?

By answering these questions, this study aimed to provide insight into the impact of PAEHR on patient care, particularly for those with mental health conditions, and to inform clinical strategies to improve the use of PAEHR in health care settings.

## Methods

A cross-sectional survey was conducted between January and February 2022. The STROBE (Strengthening the Reporting of Observational studies in Epidemiology) guidelines (Multimedia Appendix 1) for observational studies were consulted during the reporting of this cross-sectional study.

### Survey

To answer the research questions, data from the NORDeHEALTH 2022 Patient Survey were analyzed [32]. The survey was first drafted by researchers at the Norwegian Centre for E-health Research based on reviewed literature and previous Norwegian and Swedish questionnaires. It was further developed through collaboration between researchers from Norway, Sweden, Finland, and Estonia as part of the NORDeHEALTH project. The survey was designed to investigate patients’ experience with PAEHR and to identify the needs and potential challenges of online access to EHR for patients with mental health, oncology, and other health conditions. The Norwegian version of the survey consisted of 48 questions, 38 closed-ended questions and 10 free-text questions, which explored 8 thematic topics. The topics included participants’ demographic characteristics, general health status, questions about the use of the service, experience with accessing the health record, documentation and information, information security and privacy, usability, multidisciplinary teams, and functionalities of other available services in the health portal.

The survey was piloted with 4 participants who had used PAEHR from each country. Upon completion, the survey was translated into Norwegian (Multimedia Appendix 1) and made available as a pop-up for all logged-in users in the national health portal [28] for a period of 3 weeks between January 24 and February 14, 2022. The pop-up asked users if they wanted to participate in a survey about the PAEHR, and upon confirmation, they were directed to read the PAEHR before pressing “done” and proceeding to a survey page hosted on Questback (version 42; Questback) [33] platform. The survey link was only presented once per user to ensure that each patient could answer only once.

In line with the aims of this study, questions were selected from the survey to explore user characteristics; patient use of the service; and patient perceptions of perceived mistakes, omissions, and offending comments in their health records as the main outcomes. User characteristics included demographic information such as region, gender, age, highest completed education, health professional background, and current employment status. Patient use of the service was explored through questions on encouragement, reasons for use, and patient satisfaction with having access to EHR. In addition, there were questions on perceived mistakes or errors (not counting misspellings or typographical errors), omissions, and whether patients ever felt offended by something they read in the EHR. Questions on user characteristics were multiple choice, and questions regarding patient use of PAEHR and perceptions of mistakes or errors, omissions, and offended comments were mostly multiple choice and included 2 use questions scored on a 5-point Likert scale (1=strongly disagree, 2=disagree, 3=neutral, 4=agree, and 5=strongly agree). We also included an open-ended question to collect evidence of the most serious errors in EHRs perceived by mental health and somatic patients.

### Sample

The patient care type was identified by asking participants which health care services they had received in the past 2 years. The options provided in the NORDeHEALTH survey were MHC, oncology care, other health care, or no care. The participants were allowed to select ≥1 responses. Respondents were excluded from the analysis if they had not received any health care in the past 2 years. Given a very limited sample size of respondents who received only MHC and that we are focusing on users’ experience of PAEHR in an MHC context—especially considering the coexistence of mental health and somatic concerns—we categorized participants as “mental health care respondents” if they reported having received MHC in the past 2 years, regardless of whether they had concurrently received somatic health care during the same period. MHC respondents who received somatic health care during the past 2 years were therefore not identified under our analysis of “somatic health care respondents.” However, participants who exclusively received oncology or other health care within the last 2 years were identified and grouped as “somatic health care respondents.”

The MHC respondents were further classified into 3 subgroups based on the level of point of care they had received in the past 2 years: primary care, outpatient care, and hospital inpatient care. Respondents were allowed to choose multiple subgroups when answering questions about their MHC. Respondents who had selected acute care or hospital admission were automatically classified into the hospital inpatient care group. Respondents who had selected day treatment or outpatient clinic, but not acute care or hospital admission, were included in the outpatient care group. Finally, respondents who had only received MHC from a general practitioner were classified into the primary care group. Respondents who had not read their hospital mental health records at the time of the survey were not included in any of the subgroups.

### Data Analysis

Variations in demographics and perceptions concerning unexpected or offensive health records among respondents were examined in relation to different health care types (MHC and somatic health care) as well as different point-of-care levels where MHC was received (primary care, outpatient care, and hospital inpatient care). These variations were summarized by descriptive statistical analyses using SPSS statistics (version 25; IBM Corp) [34]. A Pearson chi-square test was used to explore the associations between these categorical variables. In addition, data on service use were analyzed using Excel (Microsoft Corporation) and are presented in figures representing the agreement and strong agreement responses from each patient group. The estimated response rate was calculated and reported in a previous publication [32]. As the survey was distributed to residents of Norway (regardless of health regions) who accessed their EHRs through the national health portal, it was assumed with some limitations that the selection of respondents was representative of those who used the service.

Qualitative data provided in the open-ended questions were subject to conceptual content analysis [35] using a deductive approach. The open-ended question was not mandatory and was only asked to the respondents who had answered that they had found a mistake in their EHR. They were first asked to categorize the severity of this finding and then to describe the most serious mistake. The content of comments was analyzed by 2 coders (BW and EK), following a methodology devised by Bell et al [36], “patient-reported error categories.” Comments that were irrelevant or lacked sufficient information to categorize were excluded. Both coders further discussed and refined the categories until they reached a consensus. The coding process was performed in Excel.

### Ethical Considerations

According to the Norwegian Act on Medical and Health Research §2 and §4 [37], this study did not require approval from the regional ethics committee. All data collected through the survey were anonymous. Participation was based on consent wherein each respondent could choose not to answer. The data handling procedure was approved by the data protection officer of the University Hospital of North Norway (02799).

## Results

### User Characteristics

A total of 9505 users responded to the survey in Norway. Of these, 411 (4.32%) respondents did not receive health care and were excluded from the study. Among the remaining 9094 respondents, 2008 (22.08%) MHC respondents and 7086 (77.92%) somatic health care respondents answered the survey. The region of South-Eastern Norway, which has the largest share of the population in Norway, had the highest number of responses from both MHC respondents (974/2004, 48.6%) and somatic health care respondents (3424/7069, 48.44%) groups, as shown in Table 1.

We found that female respondents made up a major proportion of both groups, particularly in the MHC group, where they represented 77.14% (1549/2008) of the respondents. Respondents of all age groups accessed their EHR online. Use of the service was higher for MHC respondents aged 15 to 44 years, whereas for somatic health care respondents, it was higher for the respondents aged between 45 and 74 years.

Of all the respondents who reported receiving MHC, 11.21% (225/2008) had no formal education or had only completed primary school, whereas 30.53% (613/2008) reported unemployment or inability to work. These proportions are twice as high compared with those of the somatic health care respondents. Approximately one-third of the respondents in both health care groups reported having a professional health education.

**Table 1:** Demographic characteristics of mental health care and somatic health care respondents.

Characteristics			Mental health care respondents, n (%)		Somatic health care respondents, n (%)		*P* value^a^
**Region (n=9073)^b^**							.28
	South and Eastern Norway	974 (48.6)		3424 (48.44)			
	Western Norway	622 (31.04)		2070 (29.28)			
	Northern Norway	327 (16.32)		1300 (18.39)			
	Central Norway	81 (4.04)		277 (3.92)			
**Gender (n=9094)^c^**							<.001
	Woman	1549 (77.14)		4451 (62.81)			
	Man	428 (21.31)		2627 (37.07)			
	Other	31 (1.54)		8 (0.11)			
**Age (y; n=9094)^c^**							<.001
	15-24	403 (20.07)		213 (3.05)			
	25-34	547 (27.24)		685 (9.67)			
	35-44	428 (21.31)		919 (12.97)			
	45-54	354 (17.63)		1516 (21.39)			
	55-64	211 (10.51)		1708 (24.1)			
	65-74	58 (2.89)		1468 (20.72)			
	75-84	5 (0.25)		548 (7.73)			
	>85	2 (0.1)		29 (0.41)			
**Education (n=9094)^c^**							<.001
	No formal education	10 (0.5)		53 (0.75)			
	Elementary school	215 (10.7)		346 (4.88)			
	Upper secondary school	674 (33.57)		1609 (22.71)			
	Higher vocational education	173 (8.61)		966 (13.63)			
	Higher education 2-4 y (bachelor)	571 (28.44)		2209 (31.17)			
	Higher education ≥4 y (master)	351 (17.48)		1810 (25.54)			
	Research doctoral degree	14 (0.7)		93 (1.31)			
**Health professional education (n=9094)^c^**							.05
	Yes	571 (28.44)		1863 (26.29)			
	No	1437 (71.56)		5223 (73.71)			
**Current employment status (n=9094)^c^**							<.001
	Full time	639 (31.82)		2993 (42.24)			
	Part-time	186 (9.26)		539 (7.61)			
	Student	314 (15.64)		179 (2.53)			
	Retired	50 (2.49)		1841 (25.98)			
	Unemployed	82 (4.08)		71 (1)			
	Not able to work	531 (26.44)		1148 (16.2)			
	None of above	206 (10.26)		315 (4.45)			

^a^*P* values comparing proportions were computed using the chi-square test. *P*<.05 indicates statistical significance.

^b^There were 2004 mental health care respondents and 7069 somatic health care respondents.

^c^There were 2008 mental health care respondents and 7086 somatic health care respondents.

### Use of the Service

Most respondents from the MHC (1343/2469, 54.39%) and somatic health care (4994/8335, 59.92%) groups reported not receiving encouragement to access their EHR (Multimedia Appendix 2). Moreover, approximately 16.77% (414/2469) of the respondents in the MHC group and 13.6% (1133/8335, 13.59%) of the respondents in the somatic health care group received encouragement from health care professionals.

**Figure 1:**
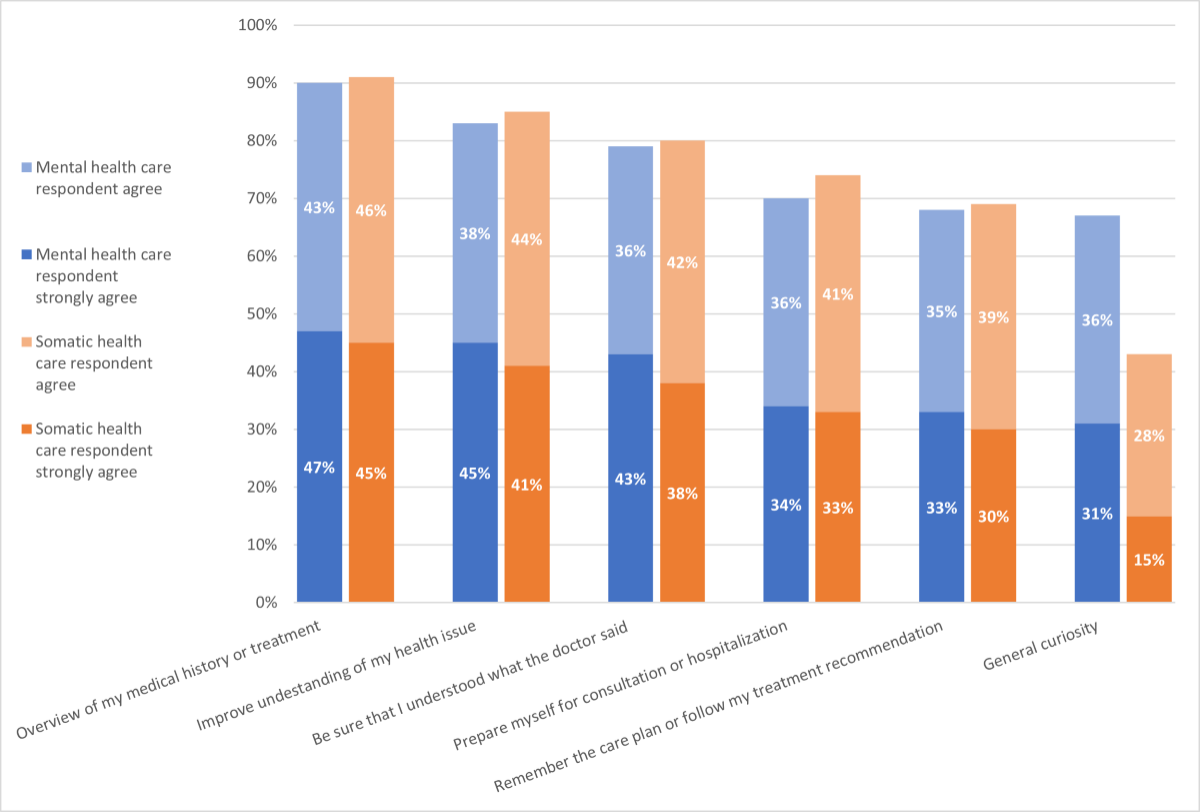
Self-reported reasons for using patient-accessible electronic health record (agree or strongly agree).

**Figure 2:**
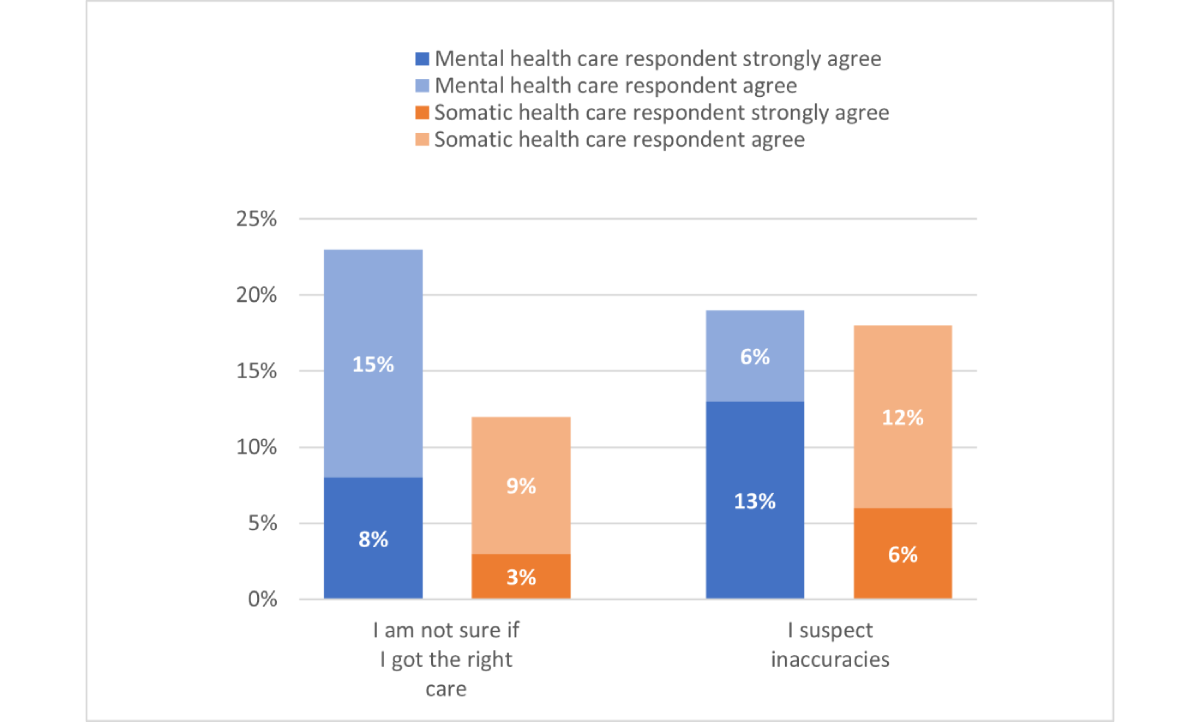
Suspected inaccuracies as a reason for using patient-accessible electronic health record (agree or strongly agree).

**Figure 3:**
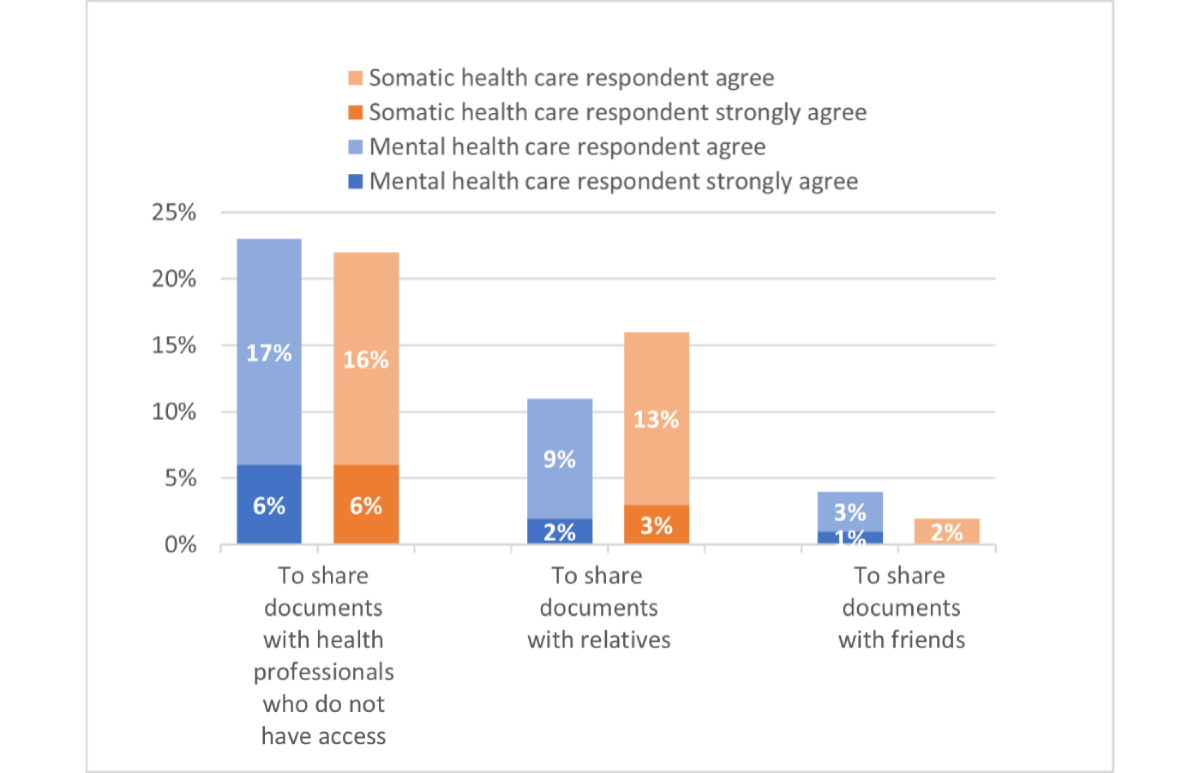
Document sharing as a reason for using the patient-accessible electronic health record (agree or strongly agree).

Reasons for PAEHR use (Multimedia Appendices 3-5 provide complete survey figures) were grouped into 3 themes based on questions’ content: *self-informing* (Figure 1), *suspecting inaccuracies* (Figure 2), and *document sharing* (Figure 3). The 2 health care groups exhibited similar tendencies in using the service, with some variations between groups. Figure 1 illustrates that a higher percentage of MHC respondents (1347/2008, 67.08%) agreed or strongly agreed that they read EHR out of general curiosity, compared with 43.03% (3049/7086) of somatic health care respondents. Similarly, MHC respondents showed a greater (percentage-wise) agreement in accessing EHR due to uncertainty about receiving the right care (446/2008, 22.21%) and identifying inaccuracies in their health records (661/2008, 32.92%) compared with somatic health care respondents (850/7086, 12% and 1275/7086, 18%, respectively; Figure 2). However, somatic health care respondents had a higher percentage (1118/7086, 15.78%) of agreement or strong agreement in using the PAEHR service for sharing documents with relatives, compared with the MHC group (222/2008, 11.05%; Figure 3).

**Figure 4:**
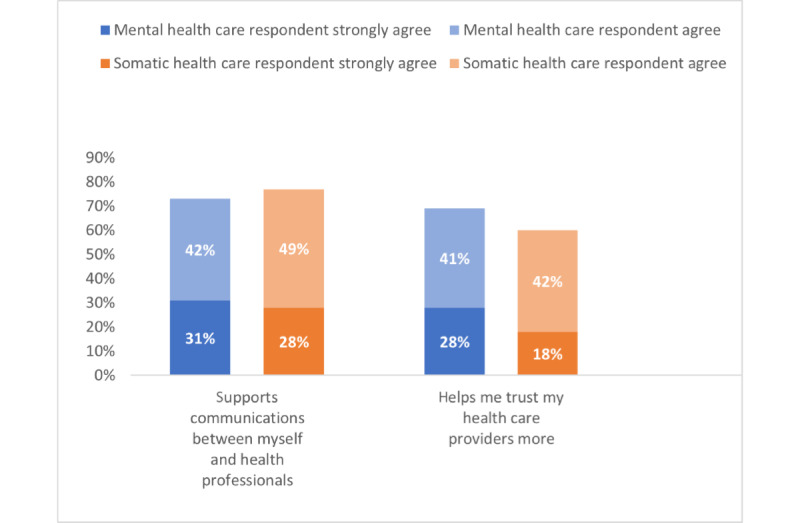
Patient experience with communication and trust by having access to electronic health record (agree or strongly agree).

About two-thirds of both health care groups expressed positive attitudes toward accessing their EHR (Multimedia Appendix 6 provides complete survey figures). Specifically, a higher proportion of MHC respondents (1385/2008, 68.97%) agreed or strongly agreed that using PAEHR increased their trust in health care professionals, compared with somatic health care respondents (4251/7086, 59.99%; Figure 4).

### Respondents’ Perceived Errors, Omissions, and Offense

In general, MHC respondents reported a higher percentage of perceived mistakes, omissions, and offended comments (*P*<.001) in their EHR compared with the somatic health care group (Table 2). Specifically, approximately 48.61% (976/2008) of the MHC respondents reported perceived mistakes and 37.75% (758/2008) reported omissions, whereas the respective percentages were both below 30% for somatic health care respondents. In addition, approximately 36.3% (729/2008) of MHC respondents reported feeling offended by something they read in their health records, whereas only 11.66% (826/7086) of somatic health care respondents reported the same.

Although most of the participants in both health care groups viewed errors or omissions as serious problems, approximately half of the respondents receiving MHC (689/1258, 54.77%) and somatic health care (1472/2976, 49.46%) did not take any action to address them. Furthermore, approximately 24.96% (314/1258) of the MHC respondents and 25.19% (750/2977) of the somatic health care respondents informed health professionals about the issue during their next visit.

**Table 2:** Respondents’ attitudes toward errors, omission, and offensive comments in health records.

Attitudes and perceptions			Mental health care respondents, n (%)		Somatic health care respondents, n (%)		*P* value^a^	
**Mistakes in your records** ^b^								<.001
	Yes	976 (48.61)		1893 (26.71)				
	No	637 (31.72)		4047 (57.11)				
	Do not know or do not remember	395 (19.67)		1146 (16.17)				
**Importance of the most serious mistakes** ^c^								<.001
	Not at all serious	90 (9.2)		264 (13.9)				
	Somewhat serious	411 (42.02)		905 (47.66)				
	Very serious	438 (44.79)		634 (33.39)				
	I am not sure	39 (3.99)		96 (5.05)				
**Omission in your records** ^b^								<.001
	Yes	758 (37.75)		1867 (26.35)				
	No	623 (31.03)		3267 (46.1)				
	Do not know or do not remember	627 (31.22)		1952 (27.55)				
**Importance of the most serious omissions** ^d^								.89
	Not at all serious	46 (6.05)		107 (5.71)				
	Somewhat serious	352 (46.3)		871 (46.5)				
	Very serious	266 (35)		640 (34.17)				
	I am not sure	96 (12.63)		255 (13.61)				
**When I found a mistake or omission** ^e^								.002
	Informed the health professional at next visit	314 (24.96)		749 (25.17)				
	Contacted the health care unit via phone	148 (11.76)		451 (15.15)				
	Did nothing	689 (54.77)		1472 (49.46)				
	Something else	107 (8.51)		304 (10.21)				
**Felt offended by something you read** ^b^								<.001
	Yes	729 (36.3)		826 (11.66)				
	No	1279 (63.7)		6260 (88.34)				

^a^*P* values comparing proportions were computed using the chi-square test. *P*<.05 indicates statistical significance.

^b^There were 2008 mental health care respondents and 7086 somatic health care respondents.

^c^There were 978 mental health care respondents 1899 for somatic health care respondents.

^d^There were 760 mental health care respondents and 1873 somatic health care respondents.

^e^There were 1258 mental health care respondents and 2976 somatic health care respondents.

### Perceptions of Persons Receiving MHC in Different Point-of-Care Levels

More persons receiving MHC in hospital inpatient care settings reported errors, omissions, and offensive comments in their hospital EHR than those in primary and outpatient care (*P*<.001; Table 3). Among those who received primary MHC, 32% (32/100) reported encountering mistakes in their hospital EHR. This proportion increased to 50.4% (422/837) for persons receiving outpatient care and reached approximately 67.7% (398/588) for those receiving hospital inpatient care. Similarly, omissions were reported by 29% (29/100) of the MHC respondents at the primary care setting, whereas 42.7% (251/588) reported them in hospital inpatient care setting. Moreover, 21% (21/100) of the respondents receiving primary MHC expressed feeling offended upon reading certain content in their health records. This percentage approximately tripled to 58.5% (344/588) among those receiving hospital inpatient care.

**Table 3:** Attitudes toward errors, omissions, and offensive comments in health records among persons receiving mental health care in different point-of-care levels.

Attitudes and perceptions		Level of point of care, respondent n (%)				*P* value^a^
		Primary care	Outpatient care	Hospital inpatient care		
**Mistakes in your records^b^**						<.001
	Yes	32 (32)	422 (50.4)	398 (67.7)		
	No	40 (40)	256 (30.6)	102 (17.3)		
	Do not know or do not remember	28 (28)	159 (19)	88 (15)		
**Importance of the most serious mistakes^c^**						<.001
	Not at all serious	8 (25)	50 (11.8)	20 (5)		
	Somewhat serious	10 (31.3)	187 (44.1)	145 (36.4)		
	Very serious	13 (40.6)	171 (40.3)	216 (54.3)		
	I am not sure	1 (3.1)	16 (3.8)	17 (4.3)		
**Omission in your records^b^**						<.001
	Yes	29 (29)	336 (40.1)	251 (42.7)		
	No	37 (37)	268 (32)	136 (23.1)		
	Do not know or do not remember	34 (34)	233 (27.8)	201 (34.2)		
**Importance of the most serious omissions^d^**						.13
	Not at all serious	3 (10.3)	22 (6.5)	11 (4.4)		
	Somewhat serious	11 (37.9)	170 (50.6)	107 (42.6)		
	Very serious	10 (34.5)	104 (30.9)	104 (41.4)		
	I am not sure	5 (17.2)	40 (11.9)	29 (11.6)		
**When I found a mistake or omission^e^**						.498
	Informed the health professional at next visit	9 (19.1)	148 (26.4)	115 (26.2)		
	Contacted the health care unit via phone	7 (14.9)	57 (10.2)	60 (13.7)		
	Did nothing	26 (55.3)	312 (55.7)	225 (51.4)		
	Something else	5 (10.6)	43 (7.7)	38 (8.7)		
**Felt offended by something you read^b^**						<.001
	Yes	21 (21)	287 (34.3)	344 (58.5)		
	No	79 (79)	550 (65.7)	244 (41.5)		

^a^*P* values comparing proportions were computed using the chi-square test. *P*<.05 indicates statistical significance.

^b^There were 100 primary care, 837 outpatient care, and 588 hospital inpatient care respondents.

^c^There were 32 primary care, 424 outpatient care, and 398 hospital inpatient care respondents.

^d^There were 29 primary care, 336 outpatient care, and 251 hospital inpatient care respondents.

^e^There were 47 primary care, 560 outpatient care, and 438 hospital inpatient care respondents.

### The Most Serious Errors Reported by MHC and Somatic Health Care Respondents

We examined free-text descriptions reported by MHC and somatic health care respondents and identified *categories of mistakes* following the methodology devised by Bell et al [36], as presented in Table 4. Overall, 26.44% (531/2088) of the MHC respondents and 24.75% (1754/7086) of the somatic health care respondents provided free-text descriptions of the most serious errors in their health records. Among those, 59 answered by MHC respondents and 249 answered by somatic health care respondents did not contain enough information to be characterized and were excluded. The final number of free-text descriptions for MHC respondents and somatic health care respondents was 472 and 1505, respectively.

Both patient groups reported similar types of mistakes in their health records. The most frequently reported mistake for MHC respondents (98/472, 22.9%) and somatic health care respondents (368/1505, 24.45%) was related to their medical history (Table 4), such as symptom details: “note from the district psychiatric centers...stated that I had been suicidal for years, which is not true at all” (MHC respondent #9426) or “[notes described] that I did not have bleeding from my rectum, but I did” (somatic health care respondent #5655). Some patients noted mistakes in dates, types, or course of operations, including documentation of operations they reported they never had. For example, “[the medical record] said I had undergone laparotomy surgery...there have been consequences for the investigations of abdominal pain afterward as (the pain) is automatically expected to be related to an operating wound I do not have” (somatic health care respondents #7525).

Communication errors were the second most common reported mistake for both MHC respondents (98/472, 22.9%) and somatic health care respondents (299/1505, 19.87%). In this category, patients described their health care professionals misinterpreting or misquoting their words during a medical visit, or professionals recorded things that did not happen during consultation. For example, 1 patient commented, “I have epilepsy and considered stopping taking medication due to side effects...communicating with the neurologist was difficult as I later found out it is said in my medical note ‘the patient wants to quit because he does not think he has epilepsy anymore’” (MHC respondent #4307). In some cases, patients reported communication failure due to language barriers, particularly when health care professionals were not fluent in Norwegian.

**Table 4:** Categories of the most serious errors reported by mental health care and somatic health care respondents.

Category		Responses and frequencies, n (%)^a^	
		Mental health care respondents (n=472)	Somatic health care respondents (n=1505)
Medical history		98 (20.8)	368 (24.5)
Communication errors		98 (20.8)	299 (19.9)
“Diagnoses” specifically mentioned		98 (20.8)	229 (15.2)
Medication and allergy reaction		61 (12.9)	173 (11.5)
Social history		36 (7.6)	119 (7.9)
**Other perceived mistakes**		30 (6.3)	168 (11.2)
	Omit something important	N/A^b^	N/A
	Copy and paste old information	N/A	N/A
Patient demographics		21 (4.4)	70 (4.7)
Wrong patient		13 (2.7)	44 (2.9)
Tests or laboratory results		12 (2.5)	47 (3.1)
Wrong side of body		11 (2.3)	44 (2.9)
Care plan		8 (1.7)	30 (2)
Exterior, doctors’, or organizational errors		8 (1.7)	49 (3.3)
Physical examination		4 (0.8)	22 (1.5)
Family history		4 (0.8)	9 (0.6)

^a^A total of 531 mental health care respondents and 1754 somatic health care respondents reported free-text errors. For mental health care respondents, 59 (11.1%) respondents did not contain sufficient information to be categorized and were excluded; frequencies were therefore calculated using 472 as a denominator. For somatic health care respondents, frequencies were calculated using 1505 as a denominator after excluding 249 (14.2%) responses lacking sufficient information.

^b^N/A: not applicable.

MHC respondents (98/472, 20.8%) reported diagnosis-related mistakes more commonly than somatic health care respondents (229/1505, 15.21%). This category included inaccurate documentation of existing diagnoses or conditions that patients did not have and diagnoses that patients had and perceived as relevant that were not recorded. For example, 1 patient said that her child’s diagnoses were wrongly set without being discussed. Some patients expressed frustration with the severe consequences, followed by erroneous diagnoses. One patient wrote, “I was diagnosed with an emotionally unstable personality disorder without any assessment...which made the whole of psychiatry turn its back on me and deny helping, even when this diagnosis is invalid” (MHC respondent #275). Several patients reported errors stemming from copy and paste of prior diagnoses.

MHC respondents reported a slightly higher frequency of mistakes about medication or allergy reactions (61/472, 12.9%) than somatic health care respondents (173/1505, 11.49%). This category included wrong or lack of medication prescription, incorrect information about active medication that patients no longer take, or wrong dose. For example, 1 patient reported not getting the right help when an acute situation arose because his physician did not write his prescription as promised. Some patients also reported mistakes related to medication allergies, including a false report of allergies that they did not have or a lack of information on their allergic reaction.

Some patients expressed satisfaction in their open-ended answers with the possibility of online access to their health records. “I would not have figured out how mistreated I was if I had not had them available” (MHC respondent #5586). However, several patients described experiences of being disrespected, labeled, or ignored when reading records. In one instance, a patient wrote, “the therapist described me in the record with personal characteristics such as disinterested and absent.... I was scared, in pain, and feeling run over; I contacted the therapist to remove this but did not even get a response” (somatic health care respondent #1732). Patients in our study also frequently reported being described by body weight (such as obese or overweight), particularly when weight-related topics were not specifically mentioned in consultation.

Despite the frequency of experienced errors, both patient groups generally found it difficult when they attempted to correct or edit recorded mistakes. One patient commented, “the threshold and possibility for correcting the medical notes is far too high” (MHC respondent #9426). Another patient believed that “patients should be asked to correct their medical records. Correcting early is cheap, easy, and not invasive” (somatic health care respondent #1234).

## Discussion

### Principal Findings

Our study showed that a significant proportion of persons with mental health and somatic concerns reported using PAEHR to stay informed about their health. In addition, some respondents reported using the service to share documents with their health care professionals and to monitor any inaccuracies in their health records. Respondents experienced that the use of PAEHR facilitated their self-informed decision-making, improved communication, and fostered trust with health care professionals, especially in the MHC settings. However, a significantly higher proportion of MHC respondents reported experiencing errors, omissions, and feeling offended by something they read in their EHR than somatic health care respondents. Similar types of errors were reported by both health care groups, with the most frequent errors related to the diagnostic process (ie, medical history, communication errors, test or laboratory results, and physical examination) and medication errors. Persons who received MHC at the hospital inpatient level had significantly higher odds of reporting errors, omissions, and feeling offended by something they read in their EHR compared with those at other levels of point of care.

Providing patients with online access to their EHR remains an ongoing discussion in the health care field, particularly in psychiatry, as it raises questions about whether it is suitable and safe for the most severely ill and the most susceptible patients [24]. Although most MHC respondents expressed a positive attitude toward transparent and accessible EHR, maintaining safety and quality with open mental health notes remains challenging. Approximately half of our MHC respondents, particularly those who received hospital inpatient care, reported perceived errors, with a quarter of them considering the errors to be very serious. These findings underscore the importance of ensuring safe and accurate documentation of mental health notes by clinicians, especially for persons with severe and chronic mental health conditions. A Delphi study [38] involving 70 respondents from mental health clinicians, patients, and informaticians predicted an increase in patients demanding changes to their clinical notes and anticipated that mental health clinicians would be less detailed or accurate in their documentation. Considering that approximately 60% of a psychiatrist’s working time in inpatient settings is unrelated to patient care [39], it is important to explore factors affecting the time and resources needed for documenting PAEHR in clinical practice. In addition, strategies to validate these errors and examine the impact of online record access on documentation practices in MHC settings need to be identified in future research.

Earlier studies showed that using standardized psychiatric diagnostic terms in documenting clinical observations may be potentially stigmatizing to patients [31,40]. Our results support this notion, as more MHC respondents resisted their diagnosis and reported diagnostic-related mistakes and feelings of violation when perceiving such mistakes compared with somatic health care respondents. The access to EHR allows patients to review stigmatized diagnostic terms such as personality disorder or schizophrenia, which can evoke distress and further damage their already fragile self-image [31]. Nevertheless, although some could argue that certain diagnostic terms in the International Statistical Classification of Diseases and Related Health Problems might be perceived as offensive, it should be noted that the International Statistical Classification of Diseases and Related Health Problems contains the contemporary shared language among MHC professionals. Therefore, a clear and specific diagnosis should not be replaced with euphemisms or vague language [31]. To mitigate the potential harm caused by access to online records, MHC respondents should be better guided and encouraged to openly discuss their diagnoses with their care providers [31,38,41]. It may also be appropriate to restrict or delay EHR access for certain patients, such as those with anorexia, who may be triggered by reading their weight and may experience further health decline. Moving forward, interventions for clinical psychiatry that use PAEHR must carefully consider the sensitivity of information, while maintaining accountability, and strive to promote a more patient-centered and empowering approach.

Respondents in both health care groups frequently expressed discomfort when being labeled using terms like “obese” or “addict.” This was especially true when these labels were used without proper context or were not discussed during a medical visit. However, labeling errors can be prevented if health care professionals empathize with their patients during interactions [42,43]. Studies have shown that health care providers’ empathy significantly affected patients’ perception of medical errors [42]. To reduce the risk of labeling errors, health care professionals can discuss terms such as obesity and addiction more empathetically [44-46] and document them using objective, factual language while considering the patient’s entire clinical presentation. For example, rather than labeling someone as a drug addict, health care professionals can use the term “substance use disorder” and provide an objective account of the patient’s medication use. As EHR becomes more transparent, health care professionals should be mindful of choosing the appropriate clinical language to avoid offending or stereotyping individuals [31,47]. One practical tip is to prepare patients for medical language they might encounter [48] and involve them in documenting their own experiences [31,47] to ensure the accuracy and reliability of EHR. This may help patients feel better understood and valued by their doctors [31,47]. It is important to acknowledge that there may still be tension between patients and physicians. However, some patients may be more sensitive owing to their mental health conditions [48]. Therefore, clinicians should learn strategies to deal with disagreement that may arise [48].

### Comparison With Prior Work

To our knowledge, no previous large-scale cross-sectional survey study has investigated and compared the extent to which patients use PAEHR in mental and somatic health care, as well as their experiences with errors, omissions, and offensive comments in EHR. Our study aligns with previous studies conducted in Norway, Sweden, and the United States [4,8,9,27,49], indicating an overall positive impact of PAEHR on user experience. However, we identified a higher proportion of patient-reported mistakes than in previous research [23,36,50]. For instance, a Finnish study conducted by Kujala et al [50] reported a lower proportion (15%) of perceived mistakes (ie, erroneous, improvised, or misunderstood) than in our study. One possible explanation for this difference could be that the Finnish study did not directly inquire about errors in the EHR but focused on the general challenges related to the patient portal.

The heightened vigilance of MHC respondents toward unexpected and offensive EHR supports previous research that raises concerns about the suitability of PAEHR for the most susceptible persons with mental illnesses, as reported by health care providers [23]. Kristiansen et al [24] found significant differences in the impact of PAEHR on patient-provider relationships and documentation practices between health care professionals in mental and somatic health care. Health care professionals in mental health settings reported to dedicate more time to documenting and explaining EHR content, as well as comforting patients during consultations after the introduction of PAEHR, compared with those in somatic health care [24]. They also exhibited a higher tendency to underreport information in the EHR and use shadow records [24]. However, it remains unclear whether these differences stem from how health personnel document in the medical record, patient sensitivity, or the suitability of these services for the most severely mentally ill patients.

### Limitations

Certain limitations should be considered when interpreting the findings from this study. First, the findings may be subject to potential response bias due to the self-reporting data used in the survey. In addition, the categorization of MHC respondents might carry a risk of selection bias, given that this category included cases with both mental and somatic issues. However, we consider this as the best way to collect experiences from a large patient care group in the current setting as only a few respondents have only received MHC. Second, as Central Norway only implemented PAEHR in May 2022 [30], it is possible that most citizens in that region do not yet have access to their EHR at the time of data collection. This could explain why we had the lowest percentage of respondents from Central Norway. Third, only an estimated response rate was available [32] as the exact number of unique users visiting the national health portal could not be retrieved by the time the data were collected. This may limit the generalizability of the results. However, we do know that all the respondents were unique, as each person was only able to reply once in this survey. Fourth, although the free-text responses from patients offered some insights, they tended to be brief and may not have fully conveyed the specific details of *why* and *how* patients experienced mistakes in their health notes. To gain a more comprehensive understanding, conducting interviews or focus groups could be a valuable approach in future research. Finally, as primary care mental health notes are currently not accessible to patients in Norway, our study only examined those patients who already had the hospital mental health notes upon visiting and receiving follow-up by primary MHC. Therefore, our findings may not fully reflect the experiences of patients receiving MHC outside the hospital setting.

### Conclusions

The study compared the experiences of respondents receiving mental and somatic health care, along with those receiving MHC at different point-of-care levels, with regard to their online access to EHR in Norway. The results indicated that most respondents in both health care groups valued accessibility and transparency provided by their EHR. MHC respondents were notably more likely to identify errors pertaining to their diagnoses and reported feeling a greater sense of violation compared with the somatic health care group. Among those receiving MHC, persons in hospital inpatient settings displayed heightened vigilance toward errors, omissions, and offensive comments in their EHR, surpassing the levels observed in other MHC settings. Although online access to EHR can provide patients with valuable information and empowerment, it also requires careful consideration when choosing clinical terms and accurate, nonjudgmental documentation concerning the unique sensitivities of patient groups. The transition toward EHR accessibility may present health care professionals with a unique opportunity to change how they think and write about patients. In the future, more studies are needed to explore the association between patient-reported mistakes and safety outcomes focusing on the specific population to yield collaboration models between clinicians and patients. Research on evaluating the impact of PAEHR over time on specific populations may also be of interest.

## Appendix

Multimedia Appendix 1 The Norwegian survey.

Multimedia Appendix 2 Encouragement of patient online access to electronic health records. Because respondents can choose multiple options, the denominator used for each group is the total number of responses within that group.

Multimedia Appendix 3 Self-information as reasons for using patient-accessible electronic health records.

Multimedia Appendix 4 Suspecting inaccuracies as reasons for using patient-accessible electronic health records.

Multimedia Appendix 5 Sharing documents as reasons for using patient-accessible electronic health records.

Multimedia Appendix 6 Patient experiences with communication and trust through access to electronic health records.

## Abbreviations

EHR: electronic health record
MHC: mental health care
PAEHR: patient-accessible electronic health record

## Data Availability

The data set analyzed during this study is available from the corresponding author upon reasonable request and following agreement from all project participants.
