# Supplementary material for: Users’ Experiences With Online Access to Electronic Health Records in Mental and Somatic Health Care: Cross-Sectional Study
Source: J Med Internet Res. 2023 Dec 25;25:e47840. doi: 10.2196/47840 (PMC10775043; doi:10.2196/47840)
Supplement: Multimedia Appendix 1 [file jmir_v25i1e47840_app1.docx]

Pasientjournal på Helsenorge

Målet med denne undersøkelsen er å finne ut hvordan du opplever å ha tilgang til din pasientjournal på internett. De fleste spørsmålene er knyttet til tjenesten Pasientjournal på Helsenorge, hvor du kan lese dokumenter fra behandling eller konsultasjon i spesialisthelsetjenesten. Ved å delta i denne undersøkelsen, bidrar du til at vi bedre forstår når og hvordan pasienter leser journalen sin på nett, og om det er noe som kan gjøres for å bedre opplevelsen av dette.

Det tar 5-10 minutter å svare.

Undersøkelsen er en del av det nordiske forskningsprosjektet NORDeHEALTH, hvor vi vil analysere og sammenligne data fra Norge, Sverige, Finland og Estland. [Nasjonalt senter for e-helseforskning](https://ehealthresearch.no/) er ansvarlig for prosjektet i Norge. Les mer om prosjektet her: <https://nordehealth.eu/>.

Ingen svar fra denne spørreundersøkelsen kommer til å videreformidles til andre eller til helsepersonell som har skrevet i din pasientjournal. Dersom du er bekymret for mulige feil i din pasientjournal, diskuter det med dem som har skrevet dokumentet.

Undersøkelsen er helt anonym, og det vil ikke være mulig å identifisere deg.

Dine svar er viktige for å kunne videreutvikle tjenesten.

1) * Hva er din alder?

|  Under 15 år |
| --- |
|  15-19 år |
|  20-24 år |
|  25-34 år |
|  35-44 år |
|  45-54 år |
|  55-64 år |
|  65-74 år |
|  75-84 år |
|  85 år eller eldre |

2) * Hvor uenig eller enig er du i følgende påstander?

|  | Helt uenig | Uenig | Litt uenig | Hverken uenig eller enig | Litt enig | Enig | Helt enig | Vet ikke |
| --- | --- | --- | --- | --- | --- | --- | --- | --- |
| Pasientjournal på Helsenorge møter mine behov. |  |  |  |  |  |  |  |  |
| Å bruke Pasientjournal på Helsenorge er en frustrerende opplevelse. |  |  |  |  |  |  |  |  |
| Pasientjournal på Helsenorge er enkel å bruke. |  |  |  |  |  |  |  |  |

3) * Kan du huske å ha hatt en spesielt positiv opplevelse med Pasientjournal på Helsenorge?

|  Ja |
| --- |
|  Nei |

**Denne informasjonen vises kun i forhåndsvisningen**

Følgende betingelser må være oppfylt for at spørsmålet skal vises for respondenten:

Dersom spørsmålet Kan du huske å ha hatt en spesielt positiv opplevelse med Pasientjournal på Helsenorge? inneholder noen av disse alternativene

- - - Ja

4) Kan du beskrive den positive opplevelsen?

5) * Kan du huske å ha hatt en spesielt negativ opplevelse med Pasientjournal på Helsenorge?

|  Ja |
| --- |
|  Nei |

**Denne informasjonen vises kun i forhåndsvisningen**

Følgende betingelser må være oppfylt for at spørsmålet skal vises for respondenten:

Dersom spørsmålet Kan du huske å ha hatt en spesielt negativ opplevelse med Pasientjournal på Helsenorge? inneholder noen av disse alternativene

- - - Ja

6) Kan du beskrive den negative opplevelsen?

7) * Hvor mange ganger tror du at du har vært inne og lest pasientjournalen din på Helsenorge de siste 12 månedene?

|  Dette er første gangen |
| --- |
|  2-9 ganger |
|  10-20 ganger |
|  Mer enn 20 ganger |

8) * Har noen av følgende oppfordret eller minnet deg om å lese pasientjournalen din på Helsenorge? Flere svar er mulig.

| Helsepersonell |
| --- |
| Skriftlig informasjon fra sykehus eller andre helseinstitusjoner |
| Familie eller venner |
| Andre pasienter |
| Nettsider, som f.eks. helsenorge.no |
| Aviser, radio, TV, Facebook etc. |
| Ingen har oppfordret meg |
| Annet |

**Denne informasjonen vises kun i forhåndsvisningen**

Følgende betingelser må være oppfylt for at spørsmålet skal vises for respondenten:

Dersom spørsmålet Har noen av følgende oppfordret eller minnet deg om å lese pasientjournalen din på Helsenorge? Flere svar er mulig. inneholder noen av disse alternativene

- - - Helsepersonell

9) * Hvilke(t) helsepersonell oppfordret eller minnet deg om å gå inn og lese dokumenter i pasientjournalen din? Flere svar er mulig.

| Lege |
| --- |
| Sykepleier |
| Psykolog |
| Fysioterapeut |
| Annet helsepersonell (f.eks. helsesekretær) |

**Denne informasjonen vises kun i forhåndsvisningen**

Følgende betingelser må være oppfylt for at spørsmålet skal vises for respondenten:

Dersom spørsmålet Har noen av følgende oppfordret eller minnet deg om å lese pasientjournalen din på Helsenorge? Flere svar er mulig. inneholder noen av disse alternativene

- - - Annet

Siden du merket av alternativet ANNET for hvordan du har blitt oppfordret til å lese pasientjournalen din på Helsenorge:

10) Kan du beskrive hvem andre eller hvordan du ble oppfordret til å lese pasientjournal (som ikke var listet opp på forrige side)?

11) * Hvor uenig eller enig er du i følgende påstander? Jeg leser pasientjournalen min på Helsenorge ...

|  | Helt uenig | Uenig | Hverken uenig eller enig | Enig | Helt enig |
| --- | --- | --- | --- | --- | --- |
| av nysgjerrighet. |  |  |  |  |  |
| for å få bedre kunnskap om min egen helse. |  |  |  |  |  |
| for å forberede meg selv til en konsultasjon eller innleggelse. |  |  |  |  |  |
| for å få oversikt over min sykdomshistorie og/eller behandling. |  |  |  |  |  |
| for å forsikre meg om at jeg forstod hva legen eller helsepersonellet sa. |  |  |  |  |  |
| for å huske behandlingsplanen eller følge anbefalingene for behandlingen min. |  |  |  |  |  |
| fordi jeg mistenker feil, mangler eller unøyaktigheter. |  |  |  |  |  |
| for å dele dokumenter med familiemedlemmer. |  |  |  |  |  |
| for å dele dokumenter med venner. |  |  |  |  |  |
| for å dele dokumenter med helsepersonell som ikke har tilgang til min pasientjournal. |  |  |  |  |  |
| fordi jeg er usikker på om jeg får riktig behandling. |  |  |  |  |  |

12) * Er det andre grunner til at du leser pasientjournalen din på Helsenorge enn de som var listet opp i forrige spørsmål?

|  Nei |
| --- |
|  Ja |

**Denne informasjonen vises kun i forhåndsvisningen**

Følgende betingelser må være oppfylt for at spørsmålet skal vises for respondenten:

Dersom spørsmålet Er det andre grunner til at du leser pasientjournalen din på Helsenorge enn de som var listet opp i forrige spørsmål? inneholder noen av disse alternativene

- - - Ja

13) Vennligst forklar grunnen(e) til at du leser pasientjournalen din (som ikke var listet opp i forrige punkt).

14) * Hvor uenig eller enig er du i følgende påstander? Å ha tilgang til pasientjournalen min ...

|  | Helt uenig | Uenig | Hverken uenig eller enig | Enig | Helt enig |
| --- | --- | --- | --- | --- | --- |
| bidrar til at jeg stoler mer på min behandler. |  |  |  |  |  |
| bidrar til bedre kommunikasjon mellom meg og helsepersonellet. |  |  |  |  |  |

15) * Har du noen gang lest noe i pasientjournalen din på Helsenorge som du mener ikke var riktig (ikke medregnet feilstavinger og grammatiske feil)?

| Ja |
| --- |
| Nei |
| Vet ikke / husker ikke |

**Denne informasjonen vises kun i forhåndsvisningen**

Følgende betingelser må være oppfylt for at spørsmålet skal vises for respondenten:

Dersom spørsmålet Har du noen gang lest noe i pasientjournalen din på Helsenorge som du mener ikke var riktig (ikke medregnet feilstavinger og grammatiske feil)? inneholder noen av disse alternativene

- - - Ja

16) * Hvor viktig var den alvorligste feilen for deg?

| Ikke viktig i det hele tatt |
| --- |
| Litt viktig |
| Svært viktig |
| Usikker |

**Denne informasjonen vises kun i forhåndsvisningen**

Følgende betingelser må være oppfylt for at spørsmålet skal vises for respondenten:

Dersom spørsmålet Har du noen gang lest noe i pasientjournalen din på Helsenorge som du mener ikke var riktig (ikke medregnet feilstavinger og grammatiske feil)? inneholder noen av disse alternativene

- - - Ja

17) Vennligst beskriv den alvorligste feilen du fant. Ikke inkluder navn eller annen personidentifiserende informasjon.

18) * Har du noen gang oppdaget at noe i pasientjournalen din mangler?

| Ja |
| --- |
| Nei |
| Vet ikke / husker ikke |

**Denne informasjonen vises kun i forhåndsvisningen**

Følgende betingelser må være oppfylt for at spørsmålet skal vises for respondenten:

Dersom spørsmålet Har du noen gang oppdaget at noe i pasientjournalen din mangler? inneholder noen av disse alternativene

- - - Ja

19) * Hvor viktig var den alvorligste mangelen du fant?

| Ikke viktig i det hele tatt |
| --- |
| Litt viktig |
| Svært viktig |
| Usikker |

**Denne informasjonen vises kun i forhåndsvisningen**

Følgende betingelser må være oppfylt for at spørsmålet skal vises for respondenten:

Dersom spørsmålet Har du noen gang oppdaget at noe i pasientjournalen din mangler? inneholder noen av disse alternativene

- - - Ja

20) Vennligst beskriv den alvorligste mangelen du fant. Ikke inkluder navn eller annen personidentifiserende informasjon.

**Denne informasjonen vises kun i forhåndsvisningen**

Følgende betingelser må være oppfylt for at spørsmålet skal vises for respondenten:

Dersom spørsmålet Har du noen gang lest noe i pasientjournalen din på Helsenorge som du mener ikke var riktig (ikke medregnet feilstavinger og grammatiske feil)? inneholder noen av disse alternativene

- - - Ja

eller

Dersom spørsmålet Har du noen gang oppdaget at noe i pasientjournalen din mangler? inneholder noen av disse alternativene

- - - Ja

21) * Gjorde du noe av følgende da du oppdaget feilen/mangelen i pasientjournalen din?

| Jeg informerte ansvarlig helsepersonell ved neste besøk. |
| --- |
| Jeg kontaktet det aktuelle helseforetaket/institusjonen via telefon. |
| Jeg gjorde ingenting. |
| Jeg gjorde noe annet. |

**Denne informasjonen vises kun i forhåndsvisningen**

Følgende betingelser må være oppfylt for at spørsmålet skal vises for respondenten:

Dersom spørsmålet Gjorde du noe av følgende da du oppdaget feilen/mangelen i pasientjournalen din? inneholder noen av disse alternativene

- - - Jeg gjorde noe annet.

22) Vennligst forklar hva du gjorde da du fant feilen eller mangelen i pasientjournalen din.

23) * Hvor lett (eller vanskelig) er det for deg å oppdage feil /mangler i pasientjournalen din?

| Svært vanskelig |
| --- |
| Vanskelig |
| Hverken vanskelig eller lett |
| Lett |
| Svært lett |

24) * Har du noen gang følt deg støtt eller fornærmet på grunn av noe du har lest i pasientjournalen din?

| Ja |
| --- |
| Nei |

**Denne informasjonen vises kun i forhåndsvisningen**

Følgende betingelser må være oppfylt for at spørsmålet skal vises for respondenten:

Dersom spørsmålet Har du noen gang følt deg støtt eller fornærmet på grunn av noe du har lest i pasientjournalen din? inneholder noen av disse alternativene

- - - Ja

25) Vennligst forklar nærmere hvorfor du har følt deg støtt eller fornærmet. Ikke inkluder navn eller annen personidentifiserende informasjon.

26) * Hvordan vil du beskrive din helsetilstand?

| Svært god |
| --- |
| God |
| Nokså god |
| Dårlig |
| Svært dårlig |
| Vet ikke / ønsker ikke å besvare |

27) * Har du i løpet av de siste to årene mottatt helsehjelp fra lege (fastlege eller spesialist) eller annet helsepersonell? Flere svar er mulig.

| Ja, for psykisk helsehjelp |
| --- |
| Ja, for kreftbehandling |
| Ja, for andre helseproblemer |
| Nei, jeg har ikke mottatt helsehjelp |

**Denne informasjonen vises kun i forhåndsvisningen**

Følgende betingelser må være oppfylt for at spørsmålet skal vises for respondenten:

Dersom spørsmålet Har du i løpet av de siste to årene mottatt helsehjelp fra lege (fastlege eller spesialist) eller annet helsepersonell? Flere svar er mulig. inneholder noen av disse alternativene

- - - Ja, for psykisk helsehjelp

28) * Har du lest dokumenter i pasientjournalen din på Helsenorge fra psykisk helsehjelp?

| Jeg har lest alt eller nesten alt. |
| --- |
| Jeg har lest noe. |
| Jeg har ikke lest noe. |

**Denne informasjonen vises kun i forhåndsvisningen**

Følgende betingelser må være oppfylt for at spørsmålet skal vises for respondenten:

Dersom spørsmålet Har du i løpet av de siste to årene mottatt helsehjelp fra lege (fastlege eller spesialist) eller annet helsepersonell? Flere svar er mulig. inneholder noen av disse alternativene

- - - Ja, for kreftbehandling

29) * Har du lest dokumenter i pasientjournalen din på Helsenorge fra kreftbehandling?

| Jeg har lest alt eller nesten alt. |
| --- |
| Jeg har lest noe. |
| Jeg har ikke lest noe. |

**Denne informasjonen vises kun i forhåndsvisningen**

Følgende betingelser må være oppfylt for at spørsmålet skal vises for respondenten:

Dersom spørsmålet Har du i løpet av de siste to årene mottatt helsehjelp fra lege (fastlege eller spesialist) eller annet helsepersonell? Flere svar er mulig. inneholder noen av disse alternativene

- - - Ja, for psykisk helsehjelp

30) * Da du mottok psykisk helsehjelp, hvor mottok du den? Flere svar er mulig.

| Hos fastlegen |
| --- |
| Dagbehandling/poliklinikk på sykehuset |
| Innleggelse ved sykehuset |
| Gjennom akutt behandling |

**Denne informasjonen vises kun i forhåndsvisningen**

Følgende betingelser må være oppfylt for at spørsmålet skal vises for respondenten:

Dersom spørsmålet Har du i løpet av de siste to årene mottatt helsehjelp fra lege (fastlege eller spesialist) eller annet helsepersonell? Flere svar er mulig. inneholder noen av disse alternativene

- - - Ja, for psykisk helsehjelp

31) * Hvor lenge har du mottatt/mottok du psykisk helsehjelp?

| Mindre enn 3 måneder |
| --- |
| Mellom 3 måneder og 1 år |
| 1-3 år |
| Mer enn 3 år |

Tverrfaglige **team**

Tverrfaglige team satt sammen av ulikt helsepersonell brukes i helsetjenesten for å samarbeide rundt behandling av spesifikke sykdommer.

32) * Har noe av din helsebehandling vært diskutert i tverrfaglige team?

| Ja |
| --- |
| Nei |
| Vet ikke |

**Denne informasjonen vises kun i forhåndsvisningen**

Følgende betingelser må være oppfylt for at spørsmålet skal vises for respondenten:

Dersom spørsmålet Har noe av din helsebehandling vært diskutert i tverrfaglige team? inneholder noen av disse alternativene

- - - Ja

33) * Ble du invitert til å delta da det tverrfaglige teamet diskuterte din helsebehandling?

| Ja |
| --- |
| Nei |

**Denne informasjonen vises kun i forhåndsvisningen**

Følgende betingelser må være oppfylt for at spørsmålet skal vises for respondenten:

Dersom spørsmålet Har noe av din helsebehandling vært diskutert i tverrfaglige team? inneholder noen av disse alternativene

- - - Ja

34) Hva var din opplevelse av det tverrfaglige teamet?

**Denne informasjonen vises kun i forhåndsvisningen**

Følgende betingelser må være oppfylt for at spørsmålet skal vises for respondenten:

Dersom spørsmålet Har noe av din helsebehandling vært diskutert i tverrfaglige team? inneholder noen av disse alternativene

- - - Ja

35) * Har du tilgang til dokumentasjon fra møtet i det tverrfaglige teamet?

| Ja |
| --- |
| Nei |
| Vet ikke |

36) * Hvor uenig eller enig er du i følgende påstander?

|  | Helt uenig | Uenig | Hverken uenig eller enig | Enig | Helt enig |
| --- | --- | --- | --- | --- | --- |
| Jeg synes at min pasientjournal på Helsenorge har et høyt sikkerhetsnivå. |  |  |  |  |  |
| Jeg stoler på at kun autorisert helsepersonell har tilgang til min pasientjournal. |  |  |  |  |  |
| Når jeg logger meg på helsenorge.no, stoler jeg på at innloggingsprosessen er sikker. |  |  |  |  |  |
| Jeg ser ingen sikkerhetsproblemer med å kopiere helseinformasjon fra min pasientjournal til andre internettbaserte programmer eller apper som Google Health, Apple Health, Facebook osv. |  |  |  |  |  |

37) * Hvor ofte klipper og limer du inn helseinformasjon fra Pasientjournal på Helsenorge til andre internettbaserte programmer eller apper (som Google Health, Apple Health, Facebook osv.)?

| Jeg har aldri gjort det. |
| --- |
| Jeg har gjort det noen få ganger (1-4 ganger). |
| Jeg har gjort det flere ganger (mer enn 5 ganger). |

38) * Har du opplevd at familiemedlemmer, venner eller andre har krevd å få tilgang til dokumenter fra Pasientjournal på Helsenorge som du ikke har ønsket å dele?

| Ja |
| --- |
| Nei |
| Vet ikke |

**Denne informasjonen vises kun i forhåndsvisningen**

Følgende betingelser må være oppfylt for at spørsmålet skal vises for respondenten:

Dersom spørsmålet Har du opplevd at familiemedlemmer, venner eller andre har krevd å få tilgang til dokumenter fra Pasientjournal på Helsenorge som du ikke har ønsket å dele? inneholder noen av disse alternativene

- - - Ja

39) * Hvem krevde å få tilgang til dokumenter som du ikke ønsket å dele?

| Et familiemedlem |
| --- |
| En venn |
| Andre |

**Denne informasjonen vises kun i forhåndsvisningen**

Følgende betingelser må være oppfylt for at spørsmålet skal vises for respondenten:

Dersom spørsmålet Hvem krevde å få tilgang til dokumenter som du ikke ønsket å dele? inneholder noen av disse alternativene

- - - Andre

40) Siden du merket av alternativet ANDRE for hvem som har krevd å få tilgang til dokumenter som du ikke ønsket å dele, ber vi om at du beskriver hvem dette er under. Ikke oppgi navn eller annen personidentifiserbar informasjon.

41) * Har du opplevd at noen har lest dokumenter fra pasientjournalen din på Helsenorge som du ikke ville dele med dem?

| Ja |
| --- |
| Nei |
| Vet ikke |

**Denne informasjonen vises kun i forhåndsvisningen**

Følgende betingelser må være oppfylt for at spørsmålet skal vises for respondenten:

Dersom spørsmålet Har du opplevd at noen har lest dokumenter fra pasientjournalen din på Helsenorge som du ikke ville dele med dem? inneholder noen av disse alternativene

- - - Ja

42) * Hvem leste dokumenter fra pasientjournalen din uten din tillatelse?

| Et helsepersonell |
| --- |
| Et familiemedlem |
| En venn |
| Andre |

**Denne informasjonen vises kun i forhåndsvisningen**

Følgende betingelser må være oppfylt for at spørsmålet skal vises for respondenten:

Dersom spørsmålet Hvem leste dokumenter fra pasientjournalen din uten din tillatelse? inneholder noen av disse alternativene

- - - Andre

43) Siden du merket av alternativet ANDRE for hvem som leste dokumenter fra pasientjournalen din uten din tillatelse, ber vi om at du beskriver hvem dette er under. Ikke oppgi navn eller annen personidentifiserbar informasjon.

44) * Anser du enkelte typer helseinformasjon som spesielt sensitiv?

| Ja |
| --- |
| Nei |

**Denne informasjonen vises kun i forhåndsvisningen**

Følgende betingelser må være oppfylt for at spørsmålet skal vises for respondenten:

Dersom spørsmålet Anser du enkelte typer helseinformasjon som spesielt sensitiv? inneholder noen av disse alternativene

- - - Ja

45) Kan du gi et eksempel på en type helseinformasjon som er spesielt sensitiv for deg?

46) * Hvor nyttig ville det ha vært for deg å ha følgende funksjoner på Helsenorge? Disse funksjonene finnes ikke på Helsenorge i dag.

|  | Ikke nyttig i det hele tatt | Ikke spesielt nyttig | Hverken ikke nyttig eller nyttig (nøytral) | Nyttig | Svært nyttig |
| --- | --- | --- | --- | --- | --- |
| Kontakte helsepersonell elektronisk for å stille spørsmål om innholdet i min pasientjournal |  |  |  |  |  |
| Påpeke feil i min pasientjournal |  |  |  |  |  |
| Skrive kommentarer i dokumenter i egen pasientjournal |  |  |  |  |  |
| Oppdatere egen helsestatus, for eksempel før neste behandling |  |  |  |  |  |
| Informere om resultater fra selv-tester eller behandling jeg har gjort hjemmefra |  |  |  |  |  |
| Dele forventninger til neste behandling/kontroll/time |  |  |  |  |  |
| Bestille eller fornye helseattester eller andre medisinske dokumenter (ikke resepter) |  |  |  |  |  |
| Få oversikt over alle mine helsekontakter, både i primær- og spesialisthelsetjenesten |  |  |  |  |  |
| Opprette et juridisk dokument som bekrefter hvilken medisinsk behandling jeg ønsker dersom jeg havner i en situasjon der jeg ikke kan kommunisere |  |  |  |  |  |
| Lese min pasientjournal fra fastlegen på nett |  |  |  |  |  |

Bakgrunnsinformasjon

47) * Hvilket fylke bor du i?

| Agder |
| --- |
| Innlandet |
| Møre og Romsdal |
| Nordland |
| Oslo |
| Rogaland |
| Vestfold og Telemark |
| Troms og Finnmark |
| Trøndelag |
| Vestland |
| Viken |
| Annet. Vennligst forklar. |

48) * Hvilket kjønn er du?

| Kvinne |
| --- |
| Mann |
| Annet |

49) * Hva er din høyeste fullførte utdanning?

| Ingen formell utdanning |
| --- |
| Grunnskole |
| Videregående skole |
| Fagskole |
| Høyere utdanning, 2-4 års varighet |
| Høyere utdanning, mer enn 4 års varighet |
| Doktorgrad |

50) * Har du helsefaglig utdanning?

| Ja |
| --- |
| Nei |

51) * Hva beskriver ditt arbeidsforhold best? Velg den mest relevante.

| Fulltid |
| --- |
| Deltid |
| Student |
| Pensjonist |
| Arbeidsledig |
| Ufør |
| Annet |

100 % fullført

Progress:

Skapt med [Questback Experience Management](https://www.questback.com/no/)

Gratis prøveabonnement – [lag en spørreundersøkelse](https://www.questback.com/lp/no/signup/?topic=quest) med Questback
