# Supplementary figures and images for: Users’ Experiences With Online Access to Electronic Health Records in Mental and Somatic Health Care: Cross-Sectional Study
Source: J Med Internet Res. 2023 Dec 25;25:e47840. doi: 10.2196/47840 (PMC10775043; doi:10.2196/47840)

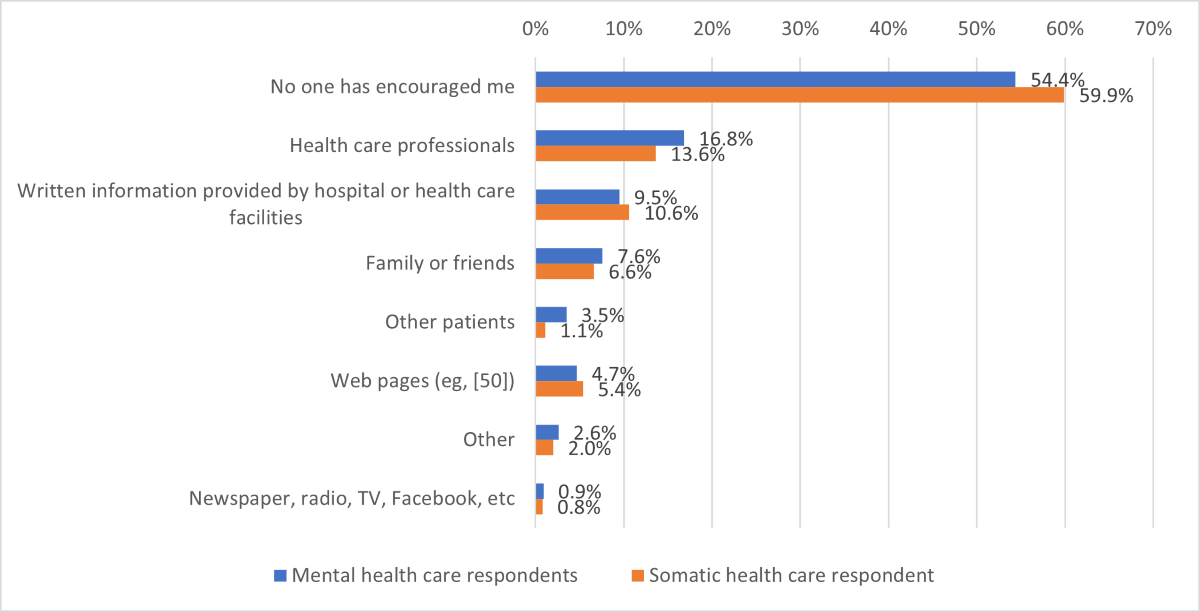

Supplement: Multimedia Appendix 2 [file jmir_v25i1e47840_app2.png]

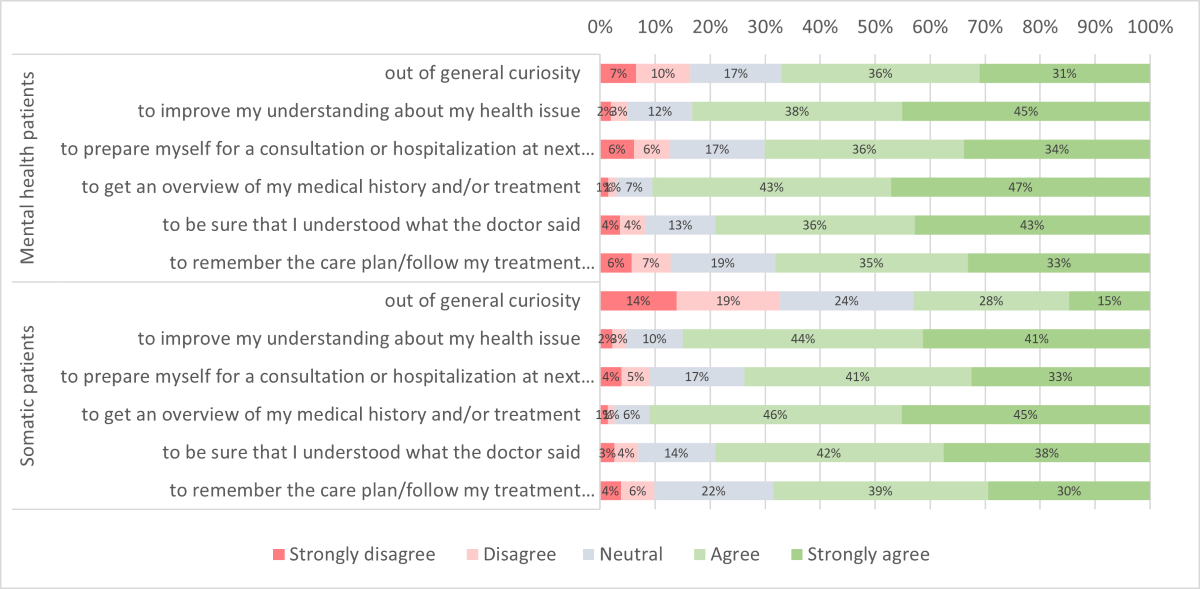

Supplement: Multimedia Appendix 3 [file jmir_v25i1e47840_app3.png]

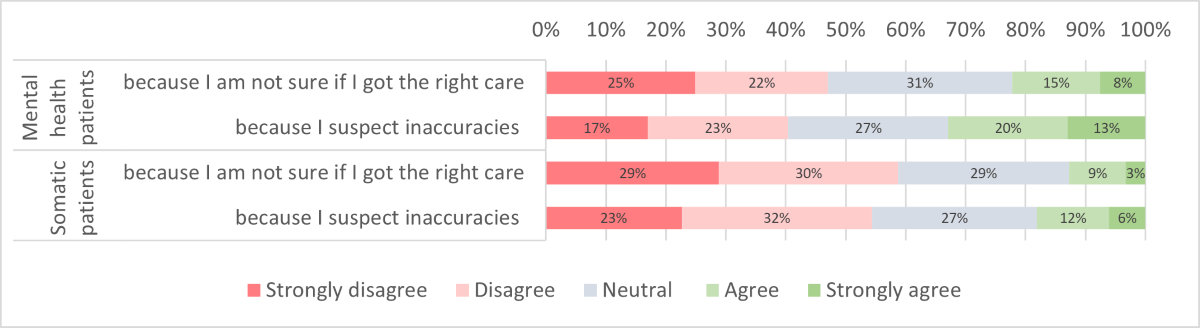

Supplement: Multimedia Appendix 4 [file jmir_v25i1e47840_app4.png]

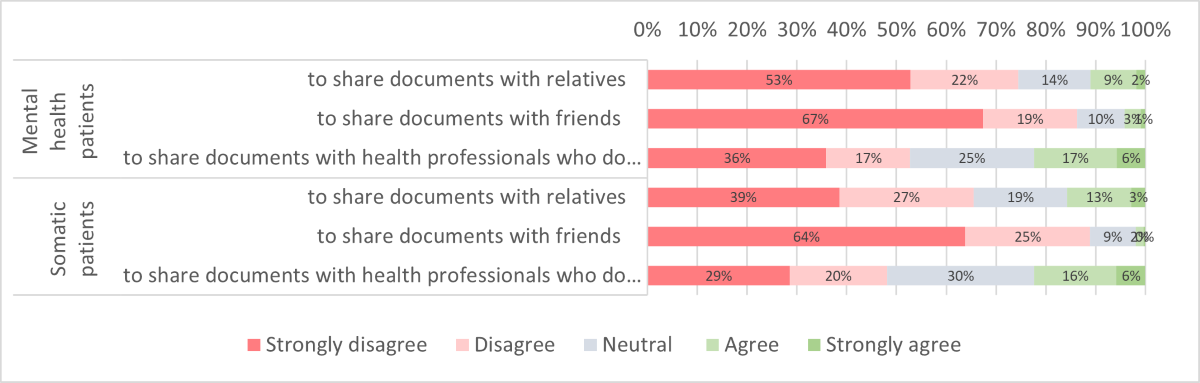

Supplement: Multimedia Appendix 5 [file jmir_v25i1e47840_app5.png]

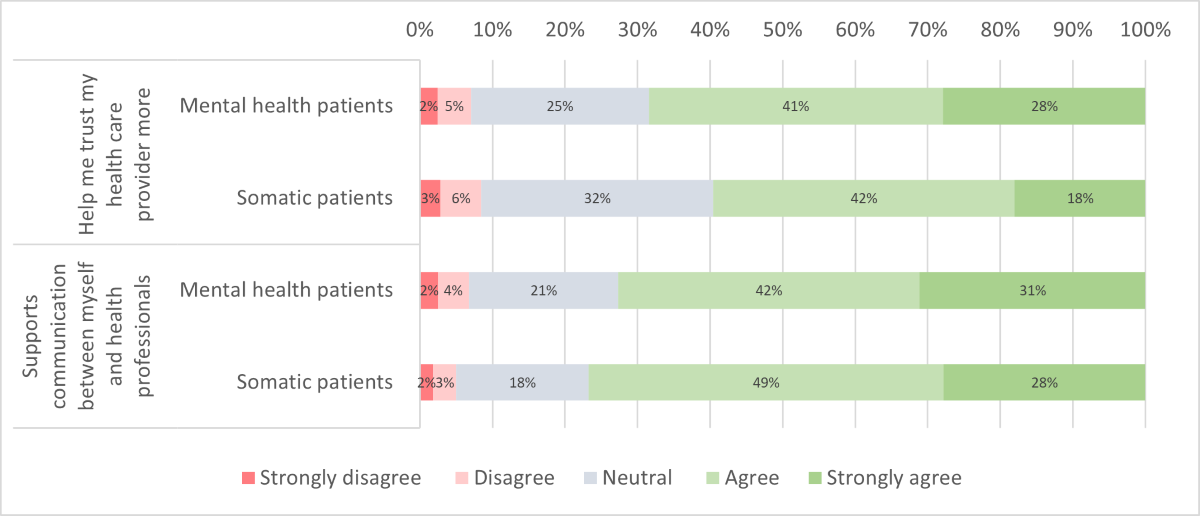

Supplement: Multimedia Appendix 6 [file jmir_v25i1e47840_app6.png]
